# Supplementary material for: Lactoferrin Inhibition of the Complex Formation between ACE2 Receptor and SARS CoV-2 Recognition Binding Domain
Source: Int J Mol Sci. 2022 May 13;23(10):5436. doi: 10.3390/ijms23105436 (PMC9141661; doi:10.3390/ijms23105436)
Supplement: Supplementary file 1 [file ijms-23-05436-s001.zip › ijms-1713628 Supplementary material.pdf]

SUPPLEMENTARY MATERIAL

BLItz SYSTEM AND ANALYTICAL MODEL

The BLItz system from Sartorius is a single-channel instrument for biolayer interferometry. It is a label-free optical technique that allows a real-time acquisition of the interference signal between white light wavelengths that are reflected by the protein layer formed on a biosensor tip. This Dip-and-Read biosensor is a disposable biosensor made from a biocompatible matrix that is uniform, non-denaturing and minimizes non-specific binding. Only molecules that bind directly to the biosensor surface are detected. The biosensor consists of an optical fiber (silicon dioxide, SiO_2) that has a highly reflecting ending internal surface; a second reflection comes from the biological layer formed on the external surface of the tip immersed in the solution. The body of the instrument consists of a mechanical arm where the biosensor is mounted. Such arm can be lifted and lowered by the user as the tip is immersed into the solution of interest. The BLItz System brings the biosensor detection surface tips directly to the sample, eliminating the need for microfluidics. This not only preserves sample integrity for reuse, but also minimizes any effects observed because of laminar flow. Any change in the number of molecules bound to the biosensor causes a shift in the interference pattern that is measured in real time. Such wavelength shift is a direct measure of the change in optical thickness of the biological layer. From the response signal, reported in relative intensity units (nm), it is possible to obtain information about the kinetics and molecular interactions of the system. To overcome the effects of diffusion on kinetic measurements, the sample plate is subject to orbital motion relative to the biosensor, introducing flow across the biosensor surface. BLI can therefore be used to measure kinetic binding constants (k_{on}, k_{off}) and the affinity constant K_D.
In a simple 1:1 binding model of a reversible reaction, the association and dissociation phases are described by a single exponential function:

$$A + B \rightleftharpoons AB$$ binding model 1:1 (S1)

$$\frac{d[AB]}{dt} = k_{on}[A][B] - k_{off}[AB]$$ during association

$$-\frac{d[AB]}{dt} = k_{off}[AB]$$ during dissociation

$$k_{on}[A][B] = k_{off}[AB] \quad \Longrightarrow \quad K_D = \frac{[A][B]}{[AB]} = \frac{k_{off}}{k_{on}}$$ at equilibrium (S2)

All curves, if left for long enough, will reach equilibrium. There is a fixed amount of receptor protein on the sensor surface, so there is a maximum possible amount of analyte protein binding at equilibrium. The equilibrium levels reached will depend on the concentration relative to the affinity constant K_D; at concentration around 100 times K_D, the equilibrium will be at a saturating concentration.
The advantage of working under PFO conditions is that time-course can be expressed with simple exponential equations (equations S3-S4), with the observed rate constant being directly related to the exponential parameter in the binding process. In the case of a simple bimolecular reaction, a plot of the observed rate constant versus the concentration of the reagent being varied will result in

a linear relationship (see equation S5). The slope and the intercept of this plot yield the apparent association and dissociation rate constants for the reaction.

$$y = y_0 + s\,(1 - \exp(-k_{obs}t)\,) \qquad \text{during association} \qquad (S3)$$

$$y = y_0 + s \exp(-k_{off}t) \qquad \text{during dissociation} \qquad (S4)$$

$$k_{obs} = k_{on}[B] + k_{off} \qquad (S5)$$

SATURATION CURVE

According to the simple system described in Supplementary material in equation S1, the saturation fraction is defined by

$$Y = \frac{[AB]}{[A]+[AB]} = \frac{[AB]}{[A_{tot}]} \qquad (S6)$$

as in the ratio between the concentration of occupied binding sites by the ligand.
Since K_D=[A][B]/[AB], substituting [AB] from this expression in equation S6 the saturation curve is

$$Y = \frac{[B]}{K_D+[B]} \qquad (S7)$$

where B is the ligand protein, the independent variable of the experiment.

For the turbidimetric assay in which LF is in solution as the analyte protein, the enhancement of absorption signal has been collected and plotted as a function of log [LF]. The increase of turbidity at 340 nm, due to protein complexes formation and hence aggregation in turbidimetric was measured. However, the observed time courses were not single exponential curves and also end points could not be estimated with certainty. Therefore, absorbance values at 340 nm at 1200 s were used as approximate end points (fig. 4a). Alternatively, only the initial rates calculated according to the classical tangent method were reported in figure 4b. Moreover, the saturation curves of figure 4 were observed to be cooperative and could not be adequately fitted to the reported equation (S7). Thus, equation S8 was used instead that explicitly takes into account a cooperativity coefficient (Hill coefficient). Thus far, the apparent thermodynamic constants could not be appropriated described as "K_D" and is referred to as *"K_{obs}".*

$$Y = \frac{[B]^n}{K_{obs}+[B]^n} \qquad (S8)$$

Overall, the values obtained for K_{obs} were comparable with both methods, as reported in the legend of fig. 4.

BLI AND TURBIDIMETRY ASSAYS

Further details are given for the reader that is not familiar with biolayer interferometry or latex nano-particles enhanced turbidimetry. In particular, from figures 2 and 3 in the text, the various steps of the measurements must be described in order to fully unveil the experimental procedure, as outlined in figure S1.

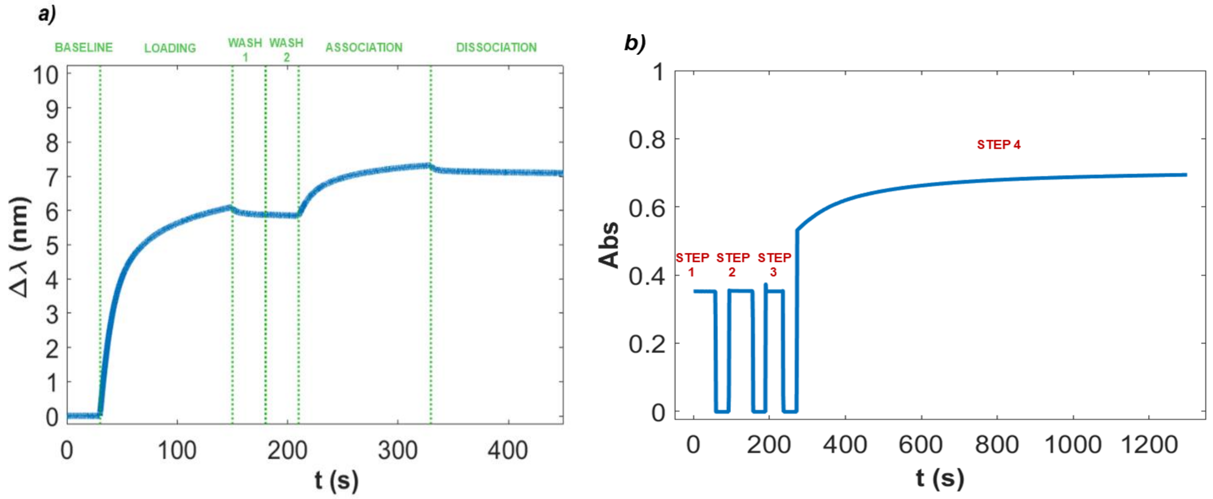

Figure S1. a) Example of time-course data acquisition in a BLI assay. Before each acquisition, a new biosensor is left hydrating in kinetic buffer for a period of 10 min; this operation is needed in order to eliminate the saccarose in which the tips are coated when packed before usage. In a BLI assay, the initial step is a "baseline step" lasting 30 s. Then the first molecule is immobilized on the biosensor during the "loading step"; the loading process takes place in a drop holder with capacity 4 µl. During all BLI experiments the concentration of the loaded protein has been maintained constant to 50 µg/ml. Before the binding step, two washing steps ("wash 1" and "wash 2") occur. The washing is done in a black tube of volume 250 µl containing the kinetic buffer provided by Sartorius (PBS with 0.02% Tween20, 0.1% BSA and 0.05% NaN_3). These steps provide baseline signals before the interaction with the analyte protein; also, between the two washing, the tube is emptied and filled with kinetic buffer again, so the biosensor tip already initialized with the receptor protein is put in a new environment cleaned of residual protein left unbound in the solution. After the washing there is the "association step" in which the initialized and cleaned biosensor is immersed in the solution containing the analyte protein (in another drop holed with capacity 4 µl). The duration of this step varies depending on the velocity of the binding between the receptor and the analyte proteins. Finally, during the "dissociation step" it is possible to observe the decreasing of the signal when dissociation happens. This step occurs again in the black tube containing 250 µl of kinetic buffer. After every assay, the equipment is cleaned with 0.5 M HCl solution. The shaker for the sample plate is set at 2200 rpm. All assays have been performed at room temperature (25°C). **b) Example of time-course acquisition in a turbidimetric assay.** Step 1 (lasting approximately 60 s) consists of acquisition of just the buffer signal. During Step 2 (lasting approximately 60 s) a suitable volume of the first protein is added, in order to monitor relevant absorbance signal caused by possible aggregation coming from the analyte protein alone. In Step 3 the inhibiting protein, lactoferrin, is injected to monitor potential aggregation with analyte protein. Finally in Step 4 the nanospheres coated with the receptor protein are added in the solution. During this final step absorbance increase

due to possible interaction with the nanospheres is observable, since such interaction is translated in an increasing signal of absorbance detected by the spectrophotometer.

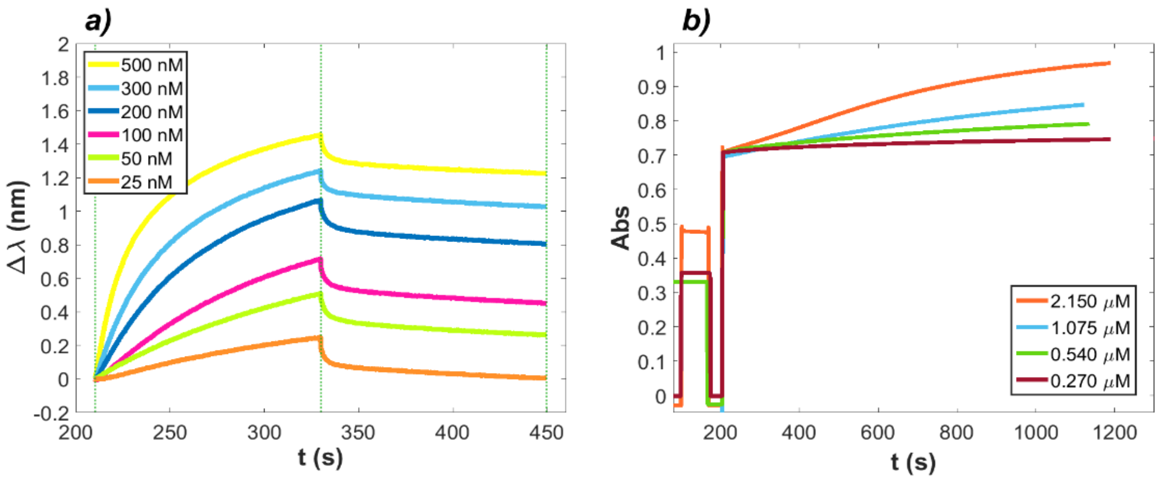

Figure S2. Time-courses experiment performed via BLI and absorption signal coming from turbidimetric assay, with the protein system RBD and ACE2 at different concentrations. a) ACE2 is in solution at decreasing concentrations while RBD is loaded on Ni-NTA biosensors. The loading step has been set to last 120 s after having verified that it was a suitable time interval for RBD to reach saturation of the biosensor surface. The vertical dashed lines indicate the duration of the binding step (120 s) and of the dissociation step (120 s). From the data fit analysis performed by BLItz software using equations S3-S4, the kinetic affinity constant is 1.96 nM. **b)** Latex nanospheres are coated with RBD protein and mixed in solution with ACE2 at decreasing concentration. The value of K_D obtained was 3.10 μM.

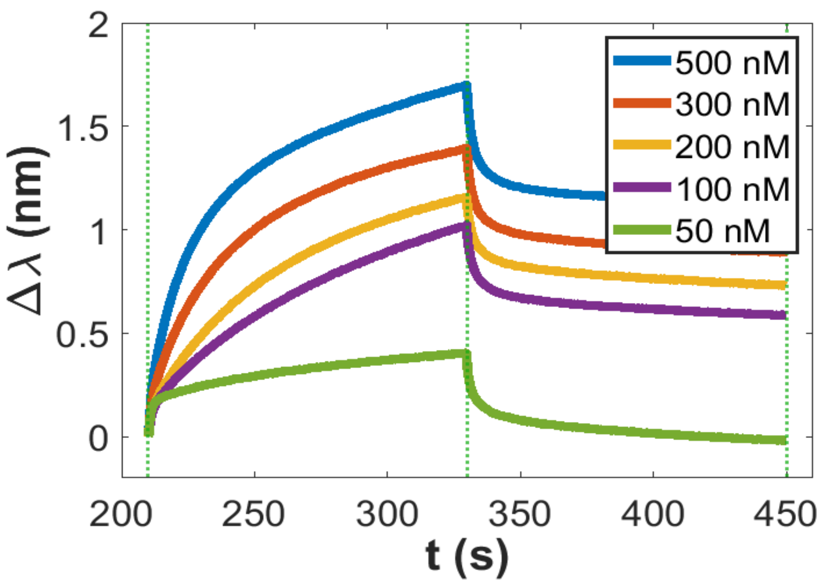

Figure S3. Signals of binding and dissociation experiment performed via BLI with the protein system RBD and ACE2 in presence of LF. RBD is loaded on anti-HIS biosensors. ACE2 and LF are present in solution as the analyte proteins. The vertical dashed lines indicate the duration of the binding step (120 s) and of the dissociation step (120 s). Concentration of LF is maintained constant at 1 µM while ACE2 is variable in the solution, at decreasing concentrations. From the data fit analysis performed by BLItz software, the obtained kinetic affinity constant is 47.87 nM.
